# Clinical relevance of circulating *ESR1* mutations during endocrine therapy for advanced hormone-dependent endometrial carcinoma

**DOI:** 10.1186/s12885-023-11559-x

**Published:** 2023-11-03

**Authors:** Aurélien Drouyer, Ludivine Beaussire, Pauline Jorda, Marianne Leheurteur, Cécile Guillemet, Anca Berghian, Dragos Georgescu, Frédéric Di Fiore, Anne Perdrix, Florian Clatot

**Affiliations:** 1 Department of Gynecology, Henri Becquerel Cancer Institute, Rouen, France; 2 Rouen Institute for Research and Innovation in Biomedicine, INSERM 1245, IRON group, Rouen, France; 3 Department of Medical Oncology, Henri Becquerel Cancer Institute, Rouen, France; 4 Department of Pathology, Henri Becquerel Cancer Institute, Rouen, France; 5 Department of Surgery, Henri Becquerel Cancer Institute, Rouen, France; 6 Department of Biopathology, Henri Becquerel Cancer Institute, Rouen, France

**Keywords:** *ESR1* mutation, Endometrial cancer, Circulating tumor DNA, Digital droplet PCR

## Abstract

**Objective:**

Endocrine therapy is frequently administered in patients with hormone dependent (HR+) metastatic endometrial cancer. *ESR1* mutations have emerged as a key mechanism of aromatase inhibitor (AI) resistance in HR + metastatic breast cancer and can be monitored using circulating tumor DNA (ctDNA). The aim of this study was to explore the incidence and clinical relevance of circulating *ESR1* mutations in patients treated by AI or megestrol acetate (M) for advanced endometrial carcinoma.

**Methodology:**

This single-center retrospective study was performed at the Henri Becquerel Center (Rouen) and looked for circulating *ESR1* gene mutations by droplet digital PCR (E380Q, L536R, Y537S, Y537N, Y537C, D538G, S463P) in patients with advanced HR + endometrial carcinoma treated between 2008 and 2020 for at least 30 days by AI or M. Analyses were performed before exposure and at progression/during endocrine therapy.

**Results:**

Twenty-two patients were included: 13 were treated with AI, 12 of whom progressed; 9 patients were treated with M, 8 of whom progressed. 68.1% of the patients had low-grade endometrial carcinoma and 54.5% had received chemotherapy in the metastatic setting. The median duration of treatment was 152 days (min 47 – max 629) with AI and 155 days (min 91-max 1297) with M. Under AI, there was no *ESR1* mutation at baseline, and one Y537C mutation at progression with a variant allele frequency (VAF) of 0.14%. Under M, one patient had a Y537C (VAF 0.2%) at baseline that disappeared during treatment. Another patient had a Y537S mutation emergence at progression after 91 days of treatment (VAF 1.83%). There was no significant difference between the circulating DNA concentration before and after hormone therapy (*p* = 0.16).

**Conclusion:**

*ESR1* mutations do not seem to be involved in the mechanisms of resistance to AI or M in HR+ endometrial cancer. The clinical relevance of their detection is not demonstrated.

**Supplementary Information:**

The online version contains supplementary material available at 10.1186/s12885-023-11559-x.

## Introduction

Endometrial cancer is the most common gynecological cancer after breast cancer. With 380,000 new cases and 90,000 deaths in 2018, it has the 4^th^ rank of women’s cancers worldwide [1].

Around 52% of endometrial cancers have progesterone receptors (PR +) and 80% have estrogen receptors (ER +) with higher rates for early stages [2]. At the metastatic stage, hormone therapy may be indicated, especially for hormone receptor-positive (HR +) tumors of low grade or slow progression. The gold standard of hormone therapy in that setting is megestrol acetate (M). Depending on comorbidities and previous endocrine therapy exposure, treatment with tamoxifen, aromatase inhibitor (AI) or LH-RH analogues may also be proposed [3]. The overall benefit of endocrine treatment for advanced endometrial cancer is moderate: with AI, the median progression free survival (PFS) is 3.9 months [4] while M provides a median PFS of 2.5 months and an overall survival (OS) of 7.6 months [5].

In HR + metastatic breast cancer, *ESR1* mutations have recently emerged as a key mechanism of AI resistance. Indeed, AI exposure in menopausal women leads to the lack of binding of estradiol to its receptor, which favors appearance of self-activating mutations. These mutations are an acquired molecular event as they are almost absent in primary tumors (< 2%), but appear in metastatic tissues in 30–50% of cases after AI exposure. *ESR1* mutations are a new prognostic factor for low survival, their occurrence can be monitored in circulating tumor DNA (ctDNA) by digital PCR (dPCR) [6]. Specific treatments are in development such as more potent or mutant-specific SERMs (Selective estrogen receptor modulators) or SERDs (selective estrogen receptor degrader) [7].

While several studies on *ESR1* mutation have been reported for breast carcinoma, very few data are available on endometrial carcinoma. Of note, a recent single observation of de novo acquired ESR1 mutation was reported under AI exposure [8]. But the importance of *ESR1* mutation in endometrial carcinoma treated by endocrine therapy is not fully understood. Interestingly, besides AI exposure, resistance to M may also be related to ESR1 mutations. Indeed, in malignant cells, M induces a modification of estrogen metabolism via the sulfatase pathway, blocking the conversion of estrone to estradiol that may lead to a high diminution of estrogen receptor binding, and favor self—activating mutations.

The aim of this study was to explore the incidence and clinical relevance of circulating *ESR1* mutations in patients treated by AI or M for advanced endometrial carcinoma.

Secondary objectives were to define the evolution of cell free DNA (cfDNA) and CA 125 levels between initiation and progression on hormone therapy; to evaluate overall survival and progression-free survival in case of circulating *ESR1* mutation and according to the hormone therapy used.

## Methods

### Patients

We retrospectively selected patients treated with AI or M for more than 30 days at the Henri Becquerel Center (Rouen) between 2008 and 2020 for endometrial cancer. Only patients with a plasma sample available at initiation of hormone therapy and at progression/during hormone therapy were included in the analysis. Patients were excluded in case of major general deterioration with a performance status > 3. The main clinical and histological characteristics of the patients were collected. In our Center, biological tests with CA 125 assays are performed regularly as part of the monitoring process. The plasma remaining after the biological analyses get stored in our plasma bank. Therefore, the plasma samples were collected prospectively but the study design and analyses were performed retrospectively. The study has been approved by the Institutional Review Board of the Henri Becquerel Center (registering order 2204B). All patients signed a consent form allowing the conservation and study of their biological samples.

### Plasma DNA extraction

Blood samples were collected in heparinized tubes before 2017 or EDTA tubes after 2017, and processed within two hours after collection with one centrifugation at 2000 g (10 min) at 4 °C before storage at -20 °C. cDNA was retrospectively extracted from 200 to 1700 μL of plasma using a QIAamp® Circulating Nucleic Acid Kit (Qiagen, Hilden, Germany). Double-stranded DNA quantification was performed by a fluorimetric method using a Quant-iT™ ds DNA HS Kit (ThermoFisher Scientific, Waltham, MA, USA).

4 ng of cDNA was preamplified with 12 cycles for heparin plasma and 9 cycles for EDTA plasma using 12.5 μL TaqMan Universal PCR Master Mix (Applied Biosystems) for heparin and 12.5 μL Q5 Hot Start High Fidelity Master Mix (New England Biolabs) for EDTA, respectively. Heparin plasma cDNA was treated with 2 μL of heparinase I bactericide beforehand to improve mutational detection by ddPCR.

### Droplet digital PCR analysis

Droplet-based dPCR (ddPCRTM) platform (Qx200® ddPCR System, Bio-rad Laboratories, Hercules, CA, USA) was used for detection of mutant cDNA in plasma samples. Custom Taqman SNP genotyping assay (Life Technologies, Carlsbad, CA) was used for the detection of *ESR1* mutations E380Q, L536R, Y537S, Y537N, Y537C, D538G and S463P.

We diluted *ESR1* mutant synthetic oligonucleotide into wild-type DNA to determine the limit of detection (LOD) of our assay. The LOD is defined as the minimum concentration of the mutant allele that can be detected from a negative control. To evaluate the LOD of our method, the allele burden was measured in 19 preamplified cfDNA extracted from healthy control heparinized plasma samples and 12 EDTA plasmas, collected in the same conditions as the patient samples. The LOD for this study was found at 0.1%.

We used QuantaSoft software (Bio-Rad, Hercules, CA, USA) which allows the calculation of the variant allele frequency (VAF) which correspond to the frequency at which the allele of a variant is found in a population, expressed in percentage. The results have been manually reviewed to allow an accurate interpretation. A sample was considered mutated if at least two ddPCR analyses found a VAF above the mutation threshold. In case of discordance of results between the duplicates, a triplicate was performed.

### Statistical analysis

As a single-center retrospective pilot study, this study is primarily descriptive and no statistical assumptions were made.

The comparison of the amount of cDNA in patients before and after initiation of hormone therapy was performed using Wilcoxon test with biostaTGV. *P*-values < 0.05 are considered significant.

## Results

### Patient characteristics and clinical outcomes

**Fig. 1:**
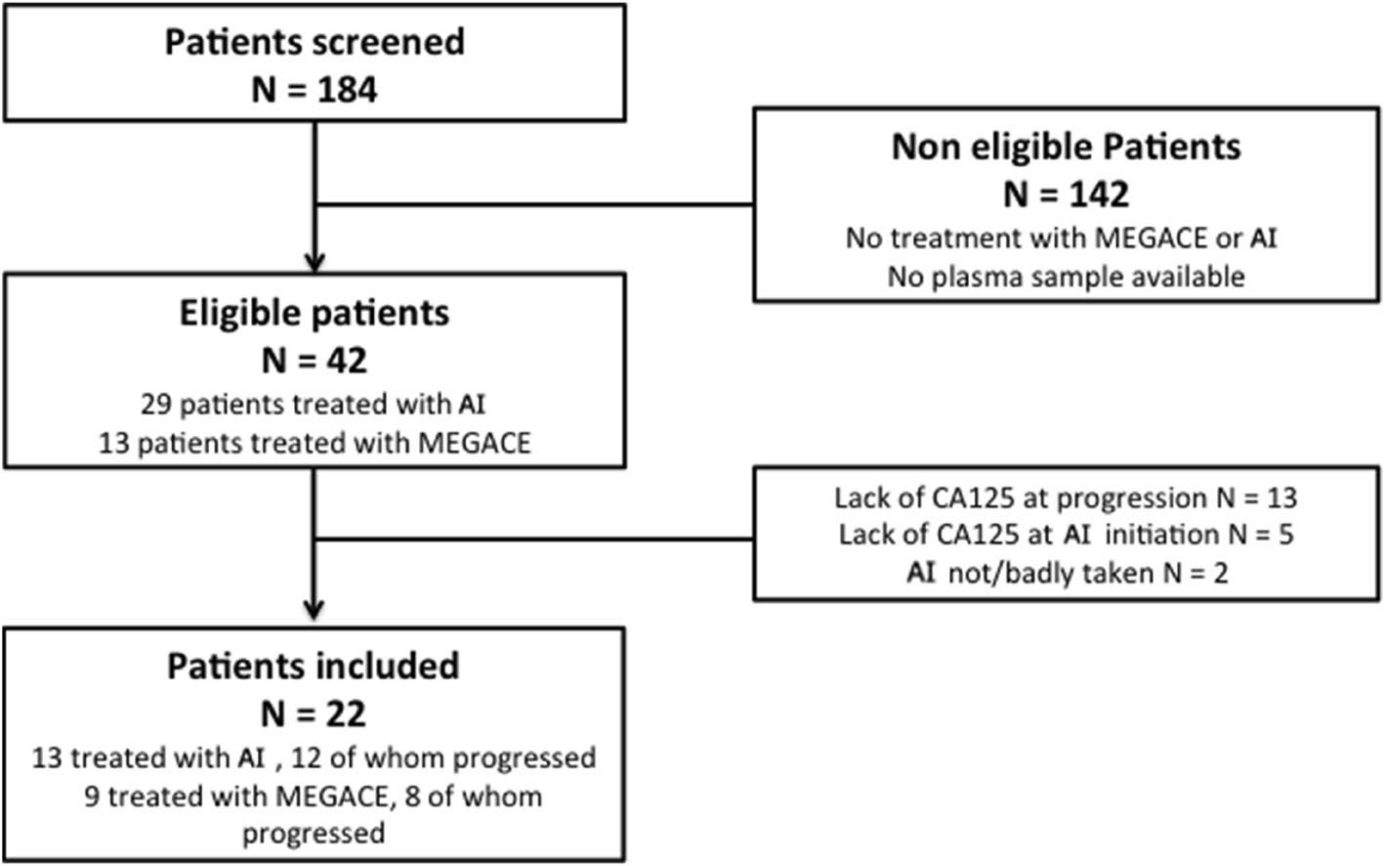
Flow chart AI: Aromatase Inhibitor

A total of 22 patients were included in this study: 13 were treated with AI, 12 of whom progressed; 9 patients were treated with M, 8 of whom progressed. A flow chart of the study is provided in Fig. 1. The main characteristics of the population are summarized in Table 1.

**Table 1:** Characteristics of the population

			**TOTAL**	**Aromatase-inhibitors**	**Megestrol acetate**
			***N* = 22**	***N* = 13**	***N* = 9**
**Median age at diagnosis** Years (min–max)			67 (49–87)	69 (49–82)	66 (60–87)
**Age at diagnosis of metastatic disease** Years (min–max)			70.4 (49–87)	72.5 (49–84)	66 (61–87)
**Grade**					
	**Low grade**		68.2% (15)	61.5% (8)	77.8% (7)
	**High grade**	Endometrioid	31.8% (7) 27.3% (6)	38.5% (5) 30.8% (4)	22.2% (2) 22.2% (2)
		Papillary serous carcinoma	4.5% (1)	7.7% (1)	-
**Stade**					
		Initially localized cancer	77.3% (17)	76.9% (10)	78.8% (7)
		Immediately metastatic	22.7% (5)	23.1% (3)	22.2% (2)
**Previous treatment**					
	**At the localized stage**				
		Surgery	63.6% (14)	61.5% (8)	66.7% (6)
		Radiotherapy	36.4% (8)	30.8% (4)	44.4% (4)
		Brachytherapy	50% (11)	53.8% (7)	44.4% (4)
		Chemotherapy	27.3% (6)	30.8% (4)	22.2% (2)
	**At the metastatic stage**				
		Chemotherapy (line number)			
		One line	40.9% (9)	15.4% (2)	77.8% (7)
		Two lines	27.3% (6)	30.8% (4)	22.2% (2)
		Megestrol acetate	4.5% (1)	7.7% (1)	-
		Anti-aromatase	4.5% (1)	-	11.1% (1)
**Median duration of hormone therapy** Days (min–max)			152 (47–1297)	152 (47–629)	155 (91–1297)
**Median BMI at diagnosis** kg/m2 (min–max)			29.6 (21.8—41.8)	30.5 (24.2–40.3)	28.3 (21.8–41.8)
**ECOG grade at diagnosis**					
		ECOG 0	50% (11)	46.2% (6)	55.6% (5)
		ECOG 1	31.8% (7)	38.5% (5)	22.2% (2)
		ECOG 2	13.7% (3)	15.4% (2)	11.1% (1)
		ECOG 3	4.5% (1)	0% (0)	11.1% (1)

*BMI* Body Mass Index, *ECOG* Eastern Cooperative Oncology Group

The median duration of treatment was 152 days with AI (min 47-max 629) and 155 days with M (min 91-max 1297). The PFS was 152 days with AI and 132 days with M. The OS was 677 with AI and 493 days with M.

Treatment was stopped for progression in 70.8% of cases and in 8.3% of cases due to poor tolerance.

### Circulating *ESR1* mutational status before exposure and at progression/during endocrine therapy

Overall, of the 22 patients, one (4.5%) circulating *ESR1* mutation was found before the initiation of hormone therapy (Y537C) and two (9.1%) circulating mutations appeared at progression (Y537C and Y537S). Cf Table 2.

**Table 2:**
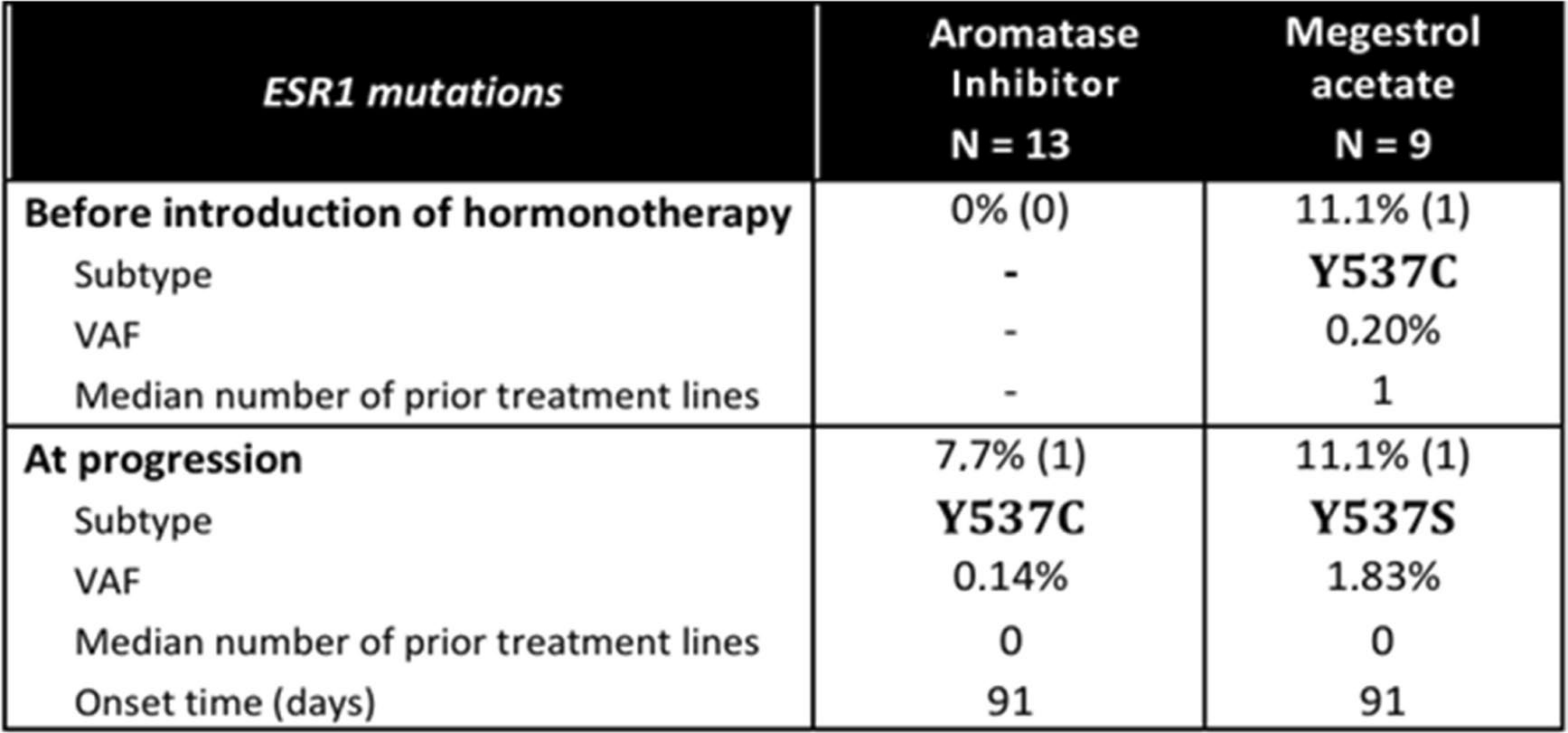
Circulating *ESR1* mutation identified under hormone therapy *VAF: Variant Allele Frequency*

More precisely, under AI, there was no *ESR1* mutation before the initiation of treatment. A Y537C mutation appeared at progression with a VAF of 0.14%. This 74-year-old patient at diagnosis had a history of an endometrioid adenocarcinoma measuring 30 × 30x20mm, grade II, with development at the uterine body and 90% myometrial invasion, initially treated by surgery and brachytherapy. Ten years later, she presented a documented pelvic and lymph node recurrence for which she was put on AI for 91 days before a change of line for progression with weekly carboplatin.

Under M, a patient presented an Y537C mutation before the initiation of hormone therapy with a VAF of 0.2%. This mutation disappeared after more than one year of treatment. This 66-year-old patient was diagnosed with a well-differentiated endometrial endometroid carcinoma, with a concomitant bone metastasis histologically proven. The patient was initially treated with chemotherapy (6 cycles of carboplatin and paclitaxel) and antalgic radiotherapy focused on the tibia. Then, a treatment with M was introduced and is continued since 2018 with an overall stability of the disease.

A Y537S mutation emerged at progression in another patient with a VAF at 1.83%. This 60-year-old patient was initially treated for a moderately differentiated invasive endometrial adenocarcinoma by surgery and then brachytherapy. Nine months later, she had a recurrence of peritoneal carcinosis with introduction of M. A new line of treatment with weekly paclitaxel was initiated after 91 days under M, due to local progression.

### cfDNA concentration

**Fig. 2:**
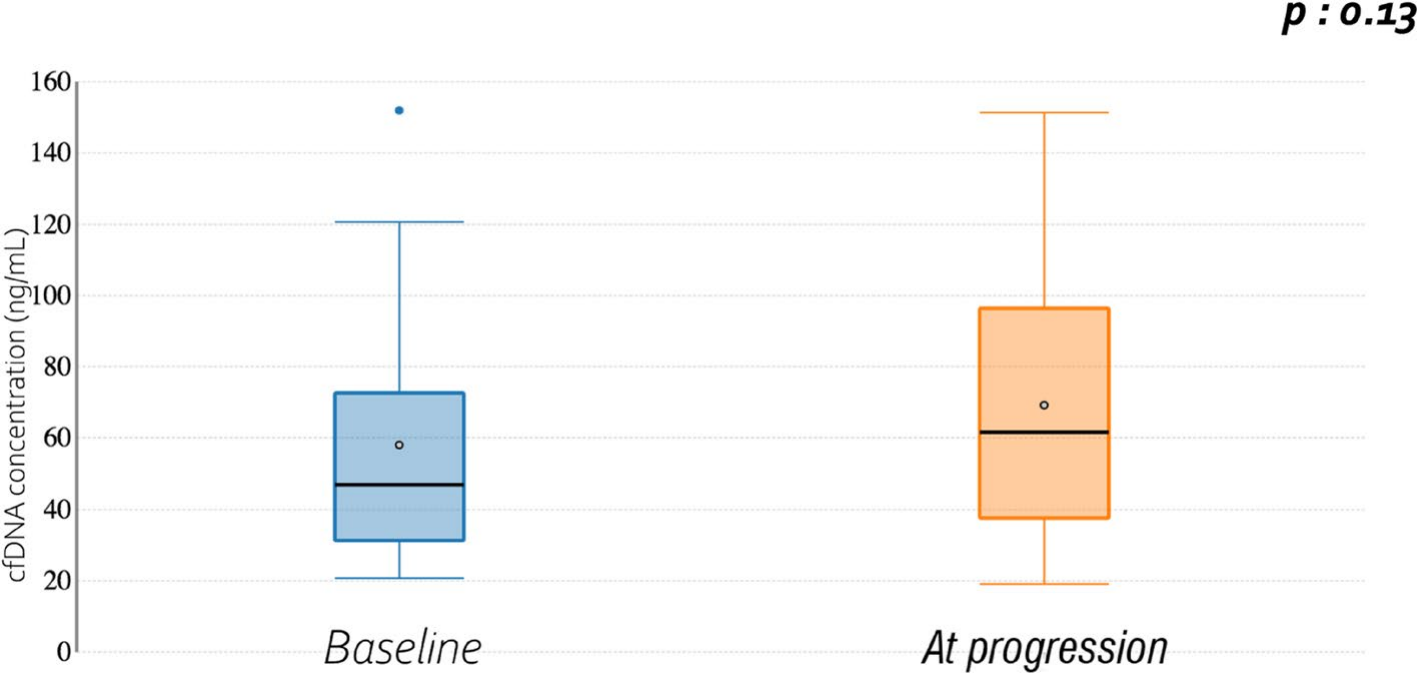
cfDNA level at hormone therapy initiation and at progression

Between initiation of hormone therapy and progression, a non-significant increase of the cfDNA levels was observed (from 46.9 ng/mL to 61.6 ng/mL, *p* = 0.131). Cf Fig. 2.

### CA125 rate

**Fig. 3:**
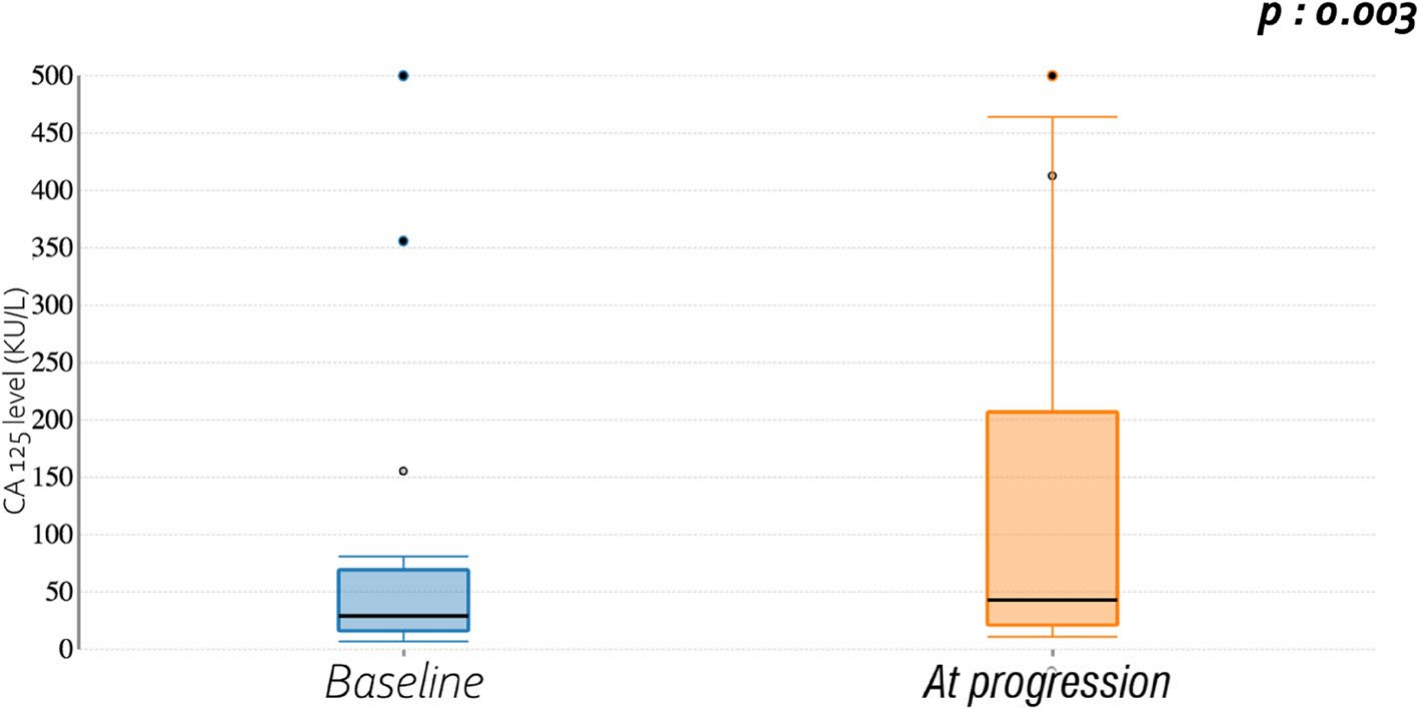
CA125 level at hormone therapy initiation and at progression

Between initiation of hormone therapy and progression, a significant increase of the CA 125 levels was observed (from 29 KU/L to 43 KU/L, *p* = 0.003). Cf Fig. 3. Of note, no correlation between the cfDNA and the CA 125 rates was observed, either at baseline or after progression on endocrine therapy (Supplementary data).

## Discussion

### Summary of main results

In this study, circulating *ESR1* mutations are a rare event since only one patient (4.5%) had a circulating *ESR1* mutation before starting hormone therapy and only two patients (9.09%) had a circulating *ESR1* mutation after progression under AI or M. In contrast, and as expected, CA 125 rate was increased at progression under endocrine therapy.

### Results in the context of published literature

The characteristics of our cohort are overall consistent with published data [9]: a majority of the patients were over 60 years old, overweight and with initially localized tumors. Of note, most of our patients presented a low grade cancer (68.2%), which contrasts with other study (33.3% of low grade carcinoma among 60 patients in Casas et al. study [10]), but this difference may be due to the selection of a population of patients treated by endocrine therapy. Regarding the treatments received, the patients included were treated between 2008 and 2020, while immunotherapy was not yet available in France (reimbursement of the pembrolizumab-lenvatinib combination in 2022).

The very low rate of circulating *ESR1* mutation at baseline in our study is in line with the histological identification of *ESR1* mutation in only 1.8% of 1034 endometrial tumors without prior treatment. Interestingly, such histological *ESR1* mutation was not associated with recurrence-free survival, age, stage and grade [9]. Concerning the response to treatment, in our study the median PFS under AI was 4.9 months, which is consistent with the 3.9 months reported in the literature [4]. Concerning the appearance of an *ESR1* mutation at progression under AI, only one case-report described an Y537S *ESR1* mutation acquired under AI, after 6 months of treatment. This mutation was found on solid biopsies, in a context of multiple lines of chemotherapy received between the initial and the second biopsy [8].

In HR + breast cancer, it is now clearly established that circulating *ESR1* mutations emergence is a mechanism of resistance to AI in the metastatic setting, with an incidence ranging from 30 to 50% depending on the series. Remarkably, these mutations are detectable between 3 and 6 months before clinical progression in 75% of patients. However, the emergence of *ESR1* mutations under AI is a late event since after three months of exposure to AI, no *ESR1* mutations are found [11]. By analogy, one can wonder if the short duration of exposure to hormone therapy in this study may explain the low number of mutations found. However, eight patients were exposed to hormone therapy for six months or more without a mutation appearing.

In this cohort, the concentration of *ESR1* mutated DNA appears to be low, with two of the three VAF right above the limit of detection (0.14% and 0.20%), and the highest VAF at 1.83%. In contrast, studies in breast carcinomas found much higher VAF, with a median VAF of 0.83% in a large study with 267 samples, and up to 44.8% [12, 13]. Thus, although the number of patients was limited, and contrary to the data available in breast cancer, our study highlights several elements suggesting that the emergence of *ESR1* mutations is not a privileged mechanism of hormone resistance in metastatic endometrial cancer: 1) Among the eight patients (36.4%) exposed to hormone therapy for at least 6 months and who progressed, only one (4.5%) had a mutation before starting hormone therapy, which disappeared upon progression. No mutations emerged on hormone therapy. 2) The very low VAF identified for 2 of the 3 patients with a mutation are rather in favour of a subclonal mechanism than a central resistance mechanism. 3) The circulating *ESR1* mutation present in one patient at the initiation of hormone therapy disappeared during treatment, which is inconsistent with a selection pressure mechanism favoring a resistance mutation.

Beyond the study of *ESR1* mutations, we also explored other circulating markers. Few data concerning cfDNA levels in endometrial cancer are published. Casas et al. reported significantly higher levels of cfDNA in patients with tumors at high risk of recurrence [10]. In contrast, Tanaka et al. did not identify significant higher levels of cfDNA in serum samples from patients with endometrial cancer (*n* = 53) than in healthy control individuals or those with benign gynecologic disorders (*n* = 24), although there was a trend (*p* = 0.09) [14]. In this study, the non-significant increase in median cfDNA level before and after hormone therapy exposure (from 46.9 ng/mL to 61.6 ng/mL, *p* = 0.13) may be related to the small number of patients and the fact that most of the tumours were of low grade, i.e. with a potential lower cfDNA release.

Because of the retrospective design of the study, the plasma samples available did not allow for a broad next generation sequencing (NGS) analysis of ctDNA and we focused on *ESR1* mutations in the context of hormone therapy resistance. However, there are data suggesting a possible interest of global tumor cDNA detection for endometrial cancers. Recent studies using ddPCR have shown that ctDNA can be detected in 41.2% of initial endometrial cancers, mainly in aggressive high-grade cancersx [10]. Moss et al. conducted a pilot study in a small cohort of 13 patients with endometrial carcinomas to investigate the clinical relevance of ctDNA to detect and monitor recurrence and progression. They showed that ctDNA analysis can detect recurrence 2.5 months before imaging and that ctDNA kinetics reflect response to treatment. The limited size of this study requires confirmation of these results. [15] Feng et al. analyzed the genome of 9 high-risk endometrial carcinoma tumor tissues and then, using specific ddPCR assays, searched for patient-specific mutations to monitor ctDNA at diagnosis and in plasma samples collected after surgery. The ctDNA appears to be useful for monitoring relapse during postoperative follow-up as a prognostic marker with a sensitivity of 100% and a specificity of 83.3%; which was superior to traditional serum tumor markers (CA125 or HE4). However, this study included a very limited number of patients. Furthermore, ctDNA was detected in only 67% of cases, mainly for FIGO stages III and IV, suggesting that ctDNA may not be sensitive enough in localized lesions but more suitable in cases with high tumor burden [16]. These data are consistent with the study by Grassi et al. in which ctDNA was detected in 60% of cases before surgery and in 27% after surgery. The detection of ctDNA before surgery is consistent with criteria of disease aggressiveness such as advanced stage, lymph node involvement, myometrial and lymphovascular invasion [17].

In our study, contrary to the analysis of cfDNA, there was a statistically significant increase in the CA125 levels between initiation of hormone therapy and progression. However, ctDNA may be more potent than CA125 in predicting progression. Indeed, the retrospective study by Pereira et al. of 44 gynecologic cancers, including 17 endometrial cancers, found an elevation of ctDNA by ddPCR six months before CA 125 elevation, with poorer survival in patients with detectable ctDNA levels at the time of surgery [18].

### Strengths and weaknesses

Our study has several limitations. First, the results should be interpreted with caution given the small number of patients included and the retrospective nature. Second, there may be a selection bias in this study due to its monocentric design. Third, the limited time of exposure to hormone therapy, together with the limited efficacy of this therapeutic class in the endometrial cancer, reduces the possibility of identifying mechanisms of acquired resistance. Finally, we do not have histological data on metastasis samples, so we cannot exclude the presence of *ESR1* mutations at the histological level, but not detectable at the circulating level. It should be noted, however, that in most solid cancers ctDNA release is frequent in the metastatic situation [19]. It therefore seems unlikely to explain the negativity of our study. One of the strong points of our study was the availability of plasma samples at the time of progression under aromatase inhibitor or megestrol acetate. Also, the methodology used for ctDNA assessment has already been validated in retrospective studies of circulating *ESR1* mutations in breast cancer [8, 11].

### Implications for practice and future research

Given the limited efficacy of hormone therapy in endometrial cancer, many mechanisms of de novo or acquired hormone resistance may exist and have to be evaluated. Different hypotheses such as the activation of cell growth signaling pathways like the PIK3A/Akt/mTOR pathway, the overexpression of EGFR or the activation of the RAS-MEK-MAPK pathway have been the subject of research in breast cancer and have led to the development of specific treatments. In breast cancer, recent clinical trials are evaluating new SERDs to counter hormone resistance acquired with AI, notably via *ESR1* mutations. Moreover, an early switch from AI to fulvestrant among patients treated by AI + palbociclib with a rising circulating *ESR1* mutation recently showed a benefit in PFS in a large phase 3 trial [6]. In endometrial cancer, there is currently no interest in testing for *ESR1* mutations prior to the introduction of these new SERDs, given the results of this study.

## Conclusion

*ESR1* mutations do not seem to be involved in the mechanisms of resistance to AI or M in HR + endometrial cancer. The clinical relevance of their detection is not demonstrated.

### Supplementary Information

**Additional file 1.**

## Data Availability

The datasets used and/or analysed during the current study are available from the corresponding author on reasonable request.

## Abbreviations

PR +: Progesterone receptors-positive
ER +: Estrogen receptors-positive
HR +: Hormone receptor-positive
M: Megestrol acetate
AI: Aromatase inhibitor
PFS: Progression free survival
SERM: Selective estrogen receptor modulators
SERD: Selective estrogen receptor degrader
ctDNA: Circulating tumor DNA
dPCR: Digital polymerase chain reaction
cfDNA: Cell free DNA
LOD: Limit of detection
VAF: Variant allele frequency
