# Supplementary material for: Clinical relevance of circulating ESR1 mutations during endocrine therapy for advanced hormone-dependent endometrial carcinoma
Source: BMC Cancer. 2023 Nov 3;23:1061. doi: 10.1186/s12885-023-11559-x (PMC10625264; doi:10.1186/s12885-023-11559-x)

SUPPLEMENTARY INFORMATION

No correlation was found between CA 125 level and cfDNA rate, either at baseline line (« J0 », r=-0.17, p=0.44) or at progression (« Prog », r=-0.02, p=0.92)


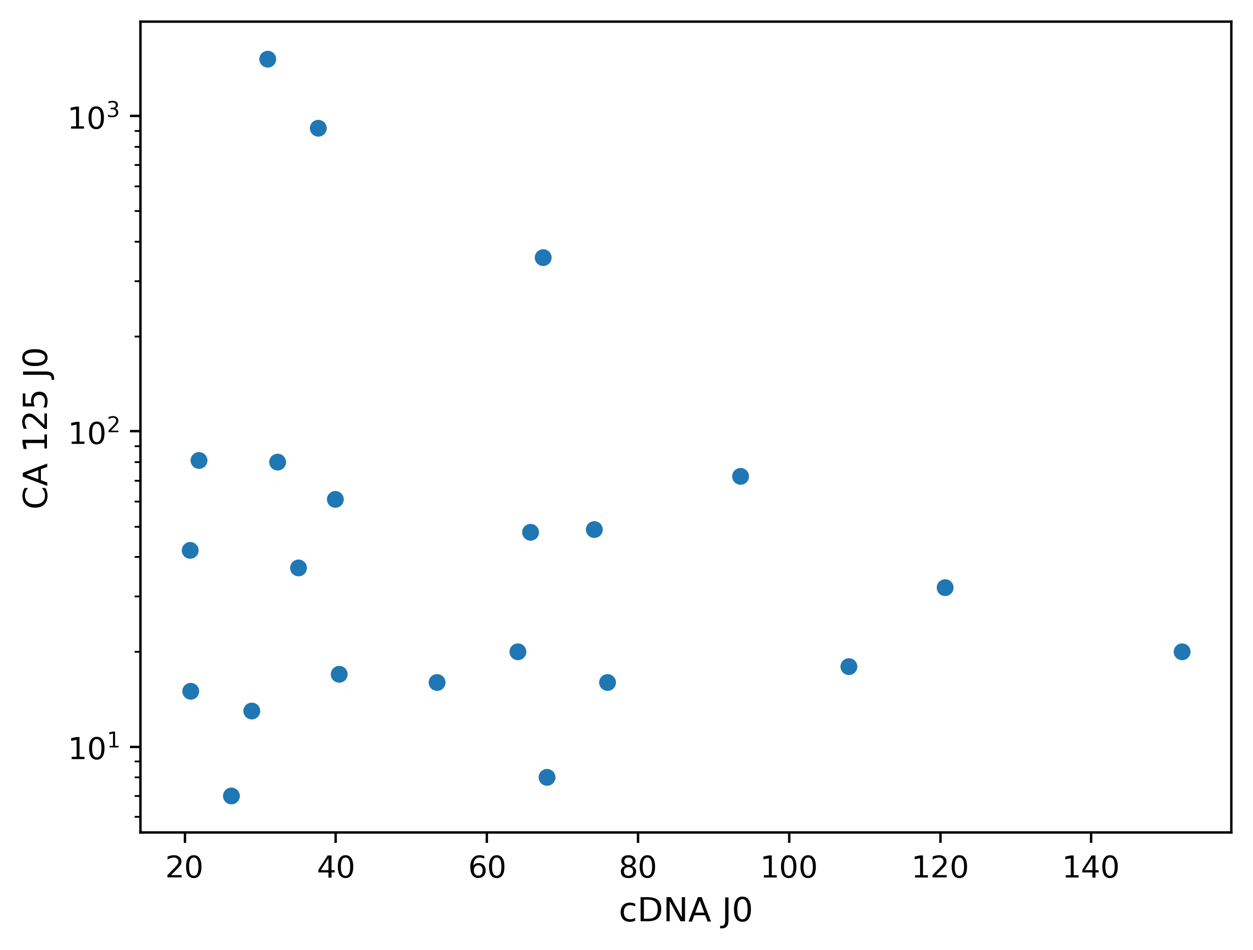


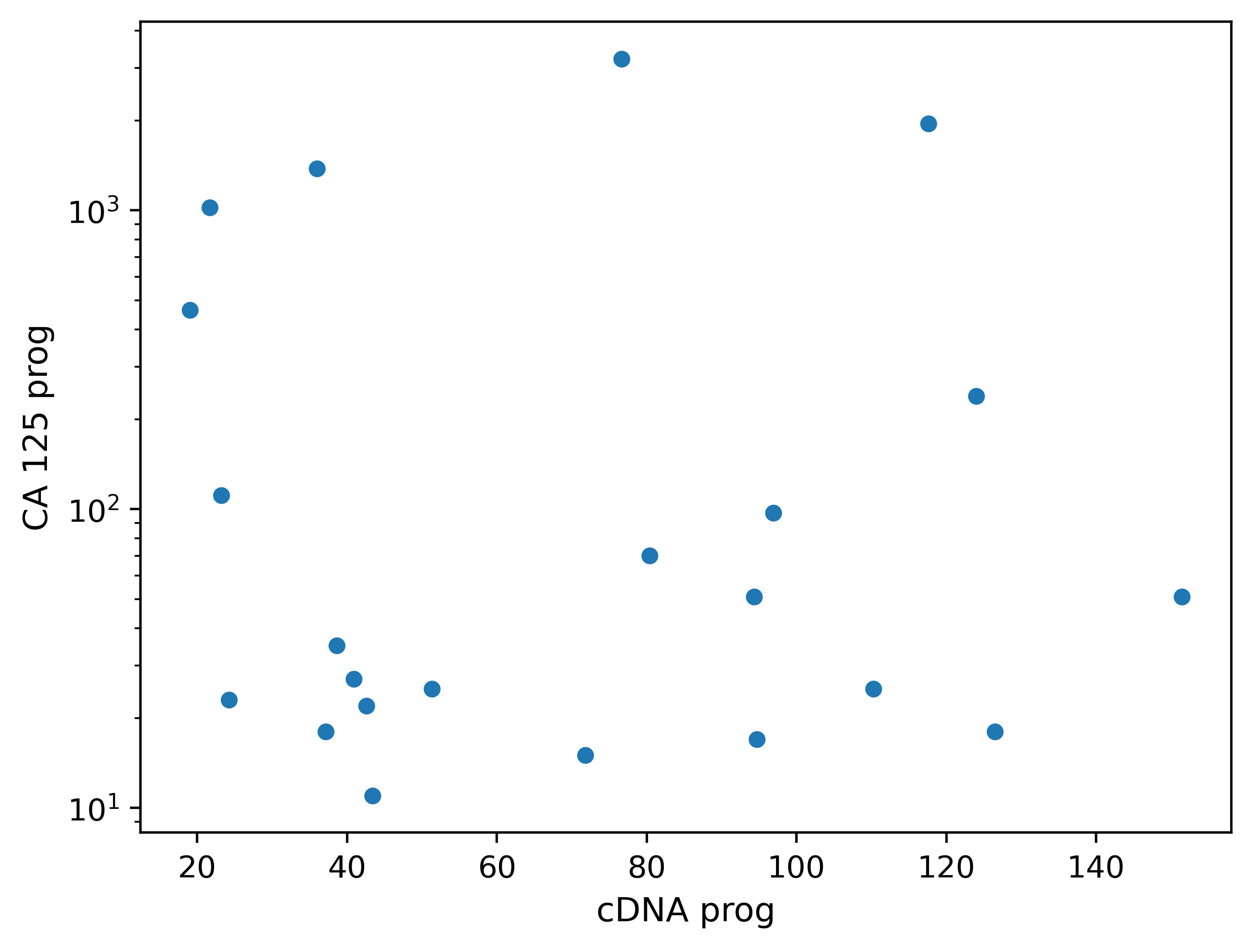

Supplement: Supplementary file 1 — Additional file 1. [file 12885_2023_11559_MOESM1_ESM.docx]
